# TXN/TXNIP signatures point to plasma cells as a potential immune hub in periodontitis based on integrated transcriptomic analysis and preliminary experimental validation

**DOI:** 10.3389/fimmu.2026.1937134

**Published:** 2026-09-30

**Authors:** Zixuan Li, Jinglun Zhang, Hongmei Zhuang, Chenyan Huang, He Zhang, Fuhua Yan

**Affiliations:** Nanjing Stomatological Hospital, Affiliated Hospital of Medical School, Institute of Stomatology, Nanjing University, Nanjing, China

**Keywords:** bioinformatics, cellular senescence, circadian rhythm, immune microenvironment, periodontitis

## Abstract

**Background:**

Periodontitis (PD) is a prevalent chronic inflammatory disease characterized by periodontal tissue destruction and alveolar bone loss. The contribution of cellular senescence- and circadian rhythm-related genes to PD progression remains incompletely defined, limiting biomarker discovery and precision therapeutic development.

**Methods:**

Publicly available PD bulk transcriptomic data, single-cell RNA-seq data, and gene sets associated with cellular senescence and circadian rhythms were integrated. Differential expression analysis (|log2FC| > 0.5, *P* < 0.05) and machine-learning strategies were used to identify key genes and establish a diagnostic model. Functional enrichment, immune infiltration, cell–cell communication, pseudotime trajectory, and drug prediction analyses were subsequently performed to characterize the biological roles and cellular specificity of these genes. Animal experiments were performed to validate the expression and cellular localization of the key genes in periodontal tissues via histologic and immunofluorescence analyses (ethics approval: No. PZW2024031).

**Results:**

*TXN* and *TXNIP* were identified as key genes. The diagnostic model based on these genes showed strong discrimination in both the training and validation sets (area under the curve > 0.7). Pathway analysis revealed that both genes were associated with antigen processing and presentation as well as with apoptosis. *TXN* was inversely related to activated B cells, whereas *TXNIP* was positively associated with immature B cells. Single-cell analysis revealed plasma cells as the principal cell subset. In PD, plasma cells changed from an immunologically isolated population to an interactive regulatory hub, communicating broadly with other cell types through the MIF signaling axis and displaying prominent functional heterogeneity and pseudotime-dependent differences.

**Conclusion:**

*TXN* and *TXNIP* may serve as candidate molecular tools for PD classification. The association of plasma cells with immune regulation highlights a potential mechanistic link between oxidative stress and immune remodeling in PD, warranting further investigation.

## 1 Introduction

Periodontitis (PD) is a chronic infectious and inflammatory disease caused by dental plaque biofilms. It is characterized primarily by the progressive destruction of periodontal supporting tissues (gingiva, periodontal ligament, and alveolar bone) (1) and severely affects the oral and systemic health of hundreds of millions of people worldwide (2). Therefore, identifying novel and effective early therapeutic targets for PD is highly important for improving long-term patient prognosis and alleviating disease burden.

Cellular senescence is an irreversible state of cell cycle arrest induced by diverse factors, including telomere shortening, DNA damage, oxidative stress, and inflammatory stimuli (3). Although senescent cells cease to divide, they remain metabolically active and profoundly impact the local microenvironment through the secretion of senescence-associated secretory phenotype (SASP) factors, including proinflammatory cytokines, chemokines, growth factors, and matrix-remodeling enzymes (4, 5). Circadian rhythms are endogenous timekeeping mechanisms evolved by organisms to adapt to the approximately 24-hour environmental cycles generated by the Earth’s rotation. They are precisely regulated by a transcription-translation feedback loop composed of a series of core clock genes, that broadly govern physiological processes such as sleep–wake cycles, hormone secretion, metabolism, and immunity (6, 7). Recently, increasing evidence has revealed a tight reciprocal regulation between cellular senescence and circadian rhythms. The disruption of clock genes can accelerate the senescence process, while senescent cells exhibit marked circadian dysregulation, thereby exacerbating tissue dysfunction and chronic inflammation (8, 9). In the context of PD, studies have confirmed the accumulation of senescent cells within periodontal tissues, demonstrating that the release of SASP factors is closely linked to PD progression and alveolar bone resorption (10). However, how the two major systems of cellular senescence and circadian rhythms act synergistically and through which molecular networks they contribute to the pathogenesis of PD remain to be systematically elucidated. Periodontal tissues are continuously exposed to microbial, inflammatory, and metabolic stress, which may disturb redox and clock-controlled immune programs and thereby connect senescence with circadian dysregulation in PD (11–13).

Building upon this background, the present study aimed to systematically investigate the roles of cellular senescence and circadian rhythm related genes in the pathogenesis of periodontitis. We hypothesized that the coordinated dysregulation of these two gene categories may contribute to the initiation and progression of periodontitis by altering the local immune microenvironment. To address this hypothesis, we analyzed publicly available transcriptomic datasets together with single–cell sequencing data to identify key molecules associated with cellular senescence and circadian rhythms and to evaluate their potential as diagnostic biomarkers for periodontitis. We further sought to characterize the specific cell types associated with these genes at the single–cell level and to explore disease related alterations in their intercellular communication networks. Through these analyses, we aimed to provide new insights into the immune regulatory mechanisms underlying periodontitis and to identify potential molecular candidates for early diagnosis and targeted intervention.

## 2 Materials and methods

### 2.1 Data sources

All bulk and single-cell transcriptomic resources were retrieved from the GEO database (https://www.ncbi.nlm.nih.gov/geo/) on August 1, 2025. GSE16134, generated on the GPL570 platform, was used as the training set and contained 241 gingival tissue samples from patients with PD and 69 gingival tissue samples from controls. In accordance with to the original publication (14), PD was defined as probing depth > 4 mm, clinical attachment loss (CAL) ≥ 3 mm, and bleeding on probing (BOP) positive; controls were defined as probing depth ≤ 4 mm, CAL ≤ 2 mm, and BOP negative. GSE173078, generated on GPL20301, served as the validation set and included 12 PD gingival tissue samples and 12 gingival tissue samples from controls. The scRNA-seq dataset GSE164241 was generated on GPL18573 and comprises gingival tissue single-cell transcriptomic data from 8 patients with PD and 13 healthy controls. According to the original publication (15), PD was defined as probing depth > 5 mm in ≥ 4 interproximal sites that were BOP positive, while controls were defined as a probing depth < 3 mm that was BOP negative; samples were 4 mm × 2 mm gingival collar biopsies. In addition, 276 cellular senescence-related genes (CSRGs) (**Supplementary Table 1**) were identified from the supplementary materials of a published paper (16), whereas 2095 circadian rhythm-related genes (CRRGs) (**Supplementary Table 1**) were identified from another published paper (17). These gene sets were downloaded on July 27, 2025. For the microarray data processing, probe-to-gene mapping was performed using the respective GPL platform annotation files. For genes with multiple probes, the probe with the highest mean expression value across all samples was selected to represent gene expression, while probes without valid gene symbols were excluded. The raw expression values were then log2-transformed (log2[x+1]) to stabilize the variance. No additional background correction or batch effect adjustment was applied, as these datasets were analyzed independently to avoid overcorrection. Missing data were not imputed, as the median expression per probe was consistently present across samples.

### 2.2 Evaluation of overall activity of senescence- and circadian rhythm-related gene sets

To evaluate whether cellular senescence- and circadian rhythm-related pathways are globally activated in PD tissues, we performed single-sample gene set enrichment analysis (ssGSEA) using the “GSVA” R package (v 1.46.0) (18). Three gene sets were scored for each sample in the training set GSE16134: the full CSRGs (n = 276), the full CRRGs (n=2095), and their intersection ACRs (n = 75). The Wilcoxon rank-sum test was used to compare the ssGSEA scores between the PD and control groups.

### 2.3 Differential expression analysis, candidate gene screening, and functional enrichment

To explore the differences in expression between PD samples and control samples, differentially expressed genes (DEGs) between PD and control samples in GSE16134 were identified with the R package “limma” (v 3.54.0) (19) using |log2Fold Change (FC)| > 0.5 and *P* < 0.05 as screening criteria. Volcano plots were generated with “ggplot2” (v 3.5.2) (20), whereas heatmaps were produced with “ComplexHeatmap” (v 2.13.0) (21). CRRGs and CSRGs were intersected using the “ggvenn” (v 0.1.9) package (22) to define aging and circadian rhythm-related genes (ACRs). DEGs subsequently overlapped with the ACR to obtain candidate genes, and the intersection was displayed as a Venn diagram.

The functional characterization of the candidate genes was performed by Gene Ontology (GO) and Kyoto Encyclopedia of Genes and Genomes (KEGG) analyses using the “clusterProfiler” (v 4.7.1) package (20), with biological process (BP), molecular function (MF), and cellular components (CC) included in the GO framework and *P* < 0.05 applied for KEGG enrichment. The 10 related pathways selected for visualization were ranked by descending P value and plotted with “GOplot” (v 1.0.2) (23). To assess protein-level interactions, candidate genes were submitted to STRING (http://www.string-db.org/) for protein–protein interaction (PPI) analysis using an integrated score ≥ 0.40. The resulting network was visualized with “circlize” (v 0.4.15) (24).

### 2.4 Identification and verification of key genes

Candidate genes were further prioritized using four machine-learning approaches. First, least absolute shrinkage and selection operator (LASSO) regression was implemented using “glmnet” (v 4.1-4) (25) with 5-fold cross-validation to reduce likelihood bias and select characteristic genes. eXtreme Gradient Boosting (XGBoost) was then applied using the R package “XGBoost” (v 2.1.1.1) (26), and features were ranked according to built-in importance scores. The “Boruta” random forest algorithm (27) was additionally used to compare real features with shadow features and identify phenotype-associated genes under p = 0.01, mcAdj = T, and maxRuns = 30. Finally, support vector machine-recursive feature elimination (SVM-RFE) was performed with the “e1071” package (28) using 10-fold cross-validation (parameter halve.above = 50), iteratively ranking gene importance and retaining the feature subset with the lowest cross-validation error. Genes shared by the four algorithms were defined as characteristic genes for downstream analyses. In GSE16134 and GSE173078, differences in the expression of characteristic genes were tested using the Wilcoxon rank-sum test. The cutoff was *P* < 0.05, and visualization was performed using “ggplot2” (v 3.5.2). Genes with significant expression changes and concordant trends across datasets were designated key genes. Receiver operating characteristic (ROC) analysis of the key genes was performed with “pROC” (v 1.18.5) (29), and the area under the curve (AUC) was used as the validation criterion.

### 2.5 Construction and evaluation of a nomogram based on key genes

To explore the predictive value of key genes for the probability of PD occurrence, a nomogram was constructed in the GSE16134 training set with “rms” (v 6.8.1) (30) based on the selected genes. Predictive calibration was evaluated by plotting a calibration curve with “regplot” (v 1.1) (31). Clinical utility was then assessed through decision curve analysis (DCA) generated by “ggDCA” (v 1.1) (32). Model discrimination was ultimately examined using ROC analysis, with AUC > 0.7 indicating favorable diagnostic performance.

### 2.6 Chromosomal and subcellular localization of key genes

To characterize the genomic and cellular contexts of the key genes, chromosomal and subcellular localization analyses were conducted based on their annotation records using “RCircos” (v 1.2.2) (33). Subcellular localization was then inferred with DeepLoc (https://services.healthtech.dtu.dk/services/DeepLoc-2.0/) to determine the cellular compartments in which the key genes may function.

### 2.7 Pathway enrichment analysis of key genes

To clarify the signaling pathways and biological functions associated with the key genes in PD, gene set enrichment analysis (GSEA) was performed using “clusterProfiler” (v 4.7.1). The MSigDB gene set “c5.go.bp.v7.5.1.entrez.gmt” was selected as the reference. The genes were ranked from high to low according to correlation coefficients. Enriched pathways were retained when the |normalized enrichment score (NES)| was > 1 and *P* was < 0.05, and the pathways with the most pronounced NES changes were selected. Intersections among gene-enriched pathways were subsequently analyzed and displayed as a heatmap. Pathway differences in the training set were further evaluated using “GSVA” (v 1.48.2) (18), which scored KEGG gene sets through the ssGSEA function; between-group differences were tested using the Wilcoxon test.

### 2.8 Immune infiltration analysis

To characterize immune microenvironmental differences between PD and control samples, ssGSEA (18) was applied to all GSE16134 samples to calculate enrichment scores for 28 immune cell types. Immune cell enrichment scores in the PD and control groups were compared using the Wilcoxon test (*P* < 0.05), and cell types with significant changes were defined as differentially enriched immune cell types. Associations between key genes and differentially enriched immune cell types were assessed in the training set by Spearman correlation analysis using “psych” (v 2.2.9) (34) with |cor| > 0.3 and *P* < 0.05 as thresholds. Correlation patterns involving immune cells and key genes with *P* < 0.05 are illustrated with a “corrplot” (v 0.95) (35).

### 2.9 Drug prediction and molecular regulatory network construction

To explore potential compounds and regulatory mechanisms, we conducted drug prediction and constructed molecular regulatory networks. Potential therapeutic compounds for PD were predicted for *TXN* and *TXNIP* using the drug–gene interaction database DsigDB (https://ngdc.cncb.ac.cn/databasecommons/database/id/4603), with *P* < 0.05 as the screening threshold. To identify upstream transcriptional regulators involved in PD-related mechanisms, NetworkAnalyst (https://www.networkanalyst.ca/NetworkAnalyst/) was used to predict TFs that interact with *TXN* or *TXNIP*. The TF-mRNA network was subsequently generated in Cytoscape (v 3.8.2) (36).

Upstream miRNAs of *TXN* and *TXNIP* were predicted using the R package “multiMiR” (37) with the PicTar and miRDB databases. Gene–miRNA pairs shared by the two prediction resources were retained as target miRNAs. miRnet (https://www.mirnet.ca/) was subsequently used to predict lncRNAs upstream of these miRNAs. *H. sapiens* was selected as the species, and Gum tissue was chosen as the project-matched tissue type. The ceRNA regulatory network was ultimately constructed in Cytoscape (v3.8.2).

### 2.10 Single-cell transcriptome analysis reveals the cell type-specific localization of key genes

Single-cell data from GSE164241 were processed using “Seurat” (v 5.0.1) (38). In terms of quality control, the percentage of retained cells was percent.mito < 15%, 200 < nFeature < 4000, and nCount_RNA < 15000. NormalizeData was then used for log normalization, followed by FindVariableFeatures to detect highly variable genes (*P* < 0.05). After ScaleData normalization, principal component analysis (PCA) was performed on the highly variable genes, and JackStraw permutation testing (*P* < 0.05) was used to assess significant principal components. ElbowPlot guided principal component selection for downstream analysis. Cell subgroups were obtained by uniform manifold approximation and projection (UMAP)-based nonlinear dimensionality reduction and clustering at a resolution of 0.4. Positive marker genes for each subgroup were identified with FindAllMarkers (min.pct = 0.25, only.pos = TRUE, logfc.threshold = 1), and CellMarker (http://xteam.xbio.top/CellMarker/) was used for cell-type annotation. To determine the cellular distribution of *TXN* and *TXNIP*, the cell type proportions in each sample were displayed in stacked plots and gene expression across subgroups was analyzed. Cell subgroups highly expressing both genes were defined as key cells.

### 2.11 Animal experiments for expression and localization validation

All animal procedures were approved by the Animal Welfare and Ethics Committee of Nanjing Agriculture University (Approval No. PZW2024031). Eight-week-old wild-type C57BL/6J male mice were obtained from Beijing Vital River Laboratory and maintained under specific pathogen-free (SPF) conditions with a 12-h light/dark cycle. Twelve mice were randomly assigned to the PD or control group (n = 6). In the PD group, 5–0 silk threads were ligated around the maxillary second molar. Three weeks post-surgery, the mice were euthanized for subsequent analyses using carbon dioxide (CO_2_) at a flow rate of 30–70% of the chamber volume per minute.

### 2.12 Histological and immunofluorescence staining

The samples were decalcified in EDTA solution for 2 weeks, followed by dehydration and paraffin embedding. Tissue sections with a thickness of 4 μm were then prepared. Hematoxylin and eosin (H&E; Sigma, USA) staining was performed to assess periodontal tissue injury. Immunofluorescence staining was conducted for TXN (1:200; A7638; ABclonal, USA), TXNIP (1:200; A9342; ABclonal, USA), and CD138 (1:200; A4174; ABclonal, USA) to evaluate their expression under PD pathological conditions.

### 2.13 Construction of the cell communication network and analysis of key cell differentiation trajectories

Cell–cell communication among clusters was evaluated using “CellChat” (v 1.6.1) (39). The aggregated communication network was calculated, and the signals derived from each cluster were visualized. Based on the communication output, the intercellular network was systematically examined to define the signaling roles of each cell type, identify the major contributing signals, and display outgoing or incoming communication patterns across cell types.

Secondary clustering was first performed on the plasma cell population to infer differentiation trajectories. The “monocle” (v 2.26.0) R package (40) was subsequently used to conduct trajectory and pseudotime analyses of key-cell clusters based on the single-cell dataset. To examine key-gene dynamics across differentiation stages, plot_genes_in_pseudotime was applied to visualize gene expression along the trajectory.

### 2.14 Analysis of functional enrichment, metabolic characteristics, and transcriptional regulation in plasma cells

For functional assessment of plasma cells in the single-cell dataset, pathway activity was calculated using the R package “GSVA” (v 1.48.2). After Z-score normalization, functional differences were displayed in a heatmap (*P* < 0.05). Gini enrichment analysis was then applied to plasma cells to define biological pathways and functions related to PD development. To further characterize the metabolic features of plasma cells, the plasma cell population was extracted from the complete single-cell dataset. Using the human KEGG metabolic pathway gene set (obtained from MSigDB), pathway activity was scored for each cell. Metabolic scores were calculated using AddModuleScore and visualized. In parallel, differential transcription factor expression across cell subgroups was analyzed using “Seurat” (v 5.0.1), and TFs highly expressed in specific subgroups were screened.

### 2.15 Statistical analysis

All bioinformatic and statistical analyses were performed using R software (version 4.3.3). Differential expression analysis between the periodontitis (PD) and control groups was conducted using the limma package, with |log2FC| > 0.5 and a nominal *P* < 0.05 used as exploratory screening criteria. This relatively inclusive threshold was selected to retain potentially relevant candidates for subsequent multistep feature selection and validation. Candidate genes were subsequently screened using LASSO, XGBoost, Boruta, and SVM-RFE and further validated in an independent GEO dataset (GSE173078). Comparisons between two independent groups, including gene expression and immune cell infiltration analyses, were performed using the Wilcoxon rank-sum test. Associations between gene expression and estimated immune cell proportions were assessed using Spearman’s rank correlation analysis, with |cor| > 0.3 and *P* < 0.05 considered significant. Comparisons of single-cell transcriptomic data were performed using the Wilcoxon rank-sum test implemented in Seurat. Unless otherwise specified, all statistical tests were two-sided, and *P* < 0.05 was considered statistically significant.

For the animal validation experiments, data are presented as the mean ± standard deviation (SD) from at least three independent biological replicates (n ≥ 3). Statistical analyses were performed using GraphPad Prism 9 (GraphPad Software, San Diego, CA, United States). Differences in protein expression between the control and periodontitis groups across different markers were analyzed using two-way ANOVA test, and *P* < 0.05 was considered statistically significant.

## 3 Results

### 3.1 Identification of candidate genes associated with cellular senescence and circadian rhythms in periodontitis

To assess whether the senescence- and circadian rhythm-related gene sets are globally represented in PD tissues, we calculated ssGSEA enrichment scores for CSRGs, CRRGs, and ACRs in the training set. There were significant differences in the enrichment scores of the three gene sets between the PD samples and the control samples (all *P* < 0.01; **Supplementary Figure 1**), indicating that these gene sets are more highly represented in PD tissues. These observations support the relevance of further investigating genes within these intersecting sets in the context of PD.

Analysis of GSE16134 revealed 1,620 DEGs associated with PD. Among these genes, 960 DEGs were upregulated and 660 were downregulated in PD (**Figures 1A, B**; **Supplementary Table 2**). CSRGs and CRRGs were curated from published literature. Integrative screening revealed 75 genes shared between the cellular senescence and circadian rhythm gene sets, defined as aging and circadian rhythm-related genes (ACRs) (**Figure 1C**; **Supplementary Table 3**).

**Figure 1:**
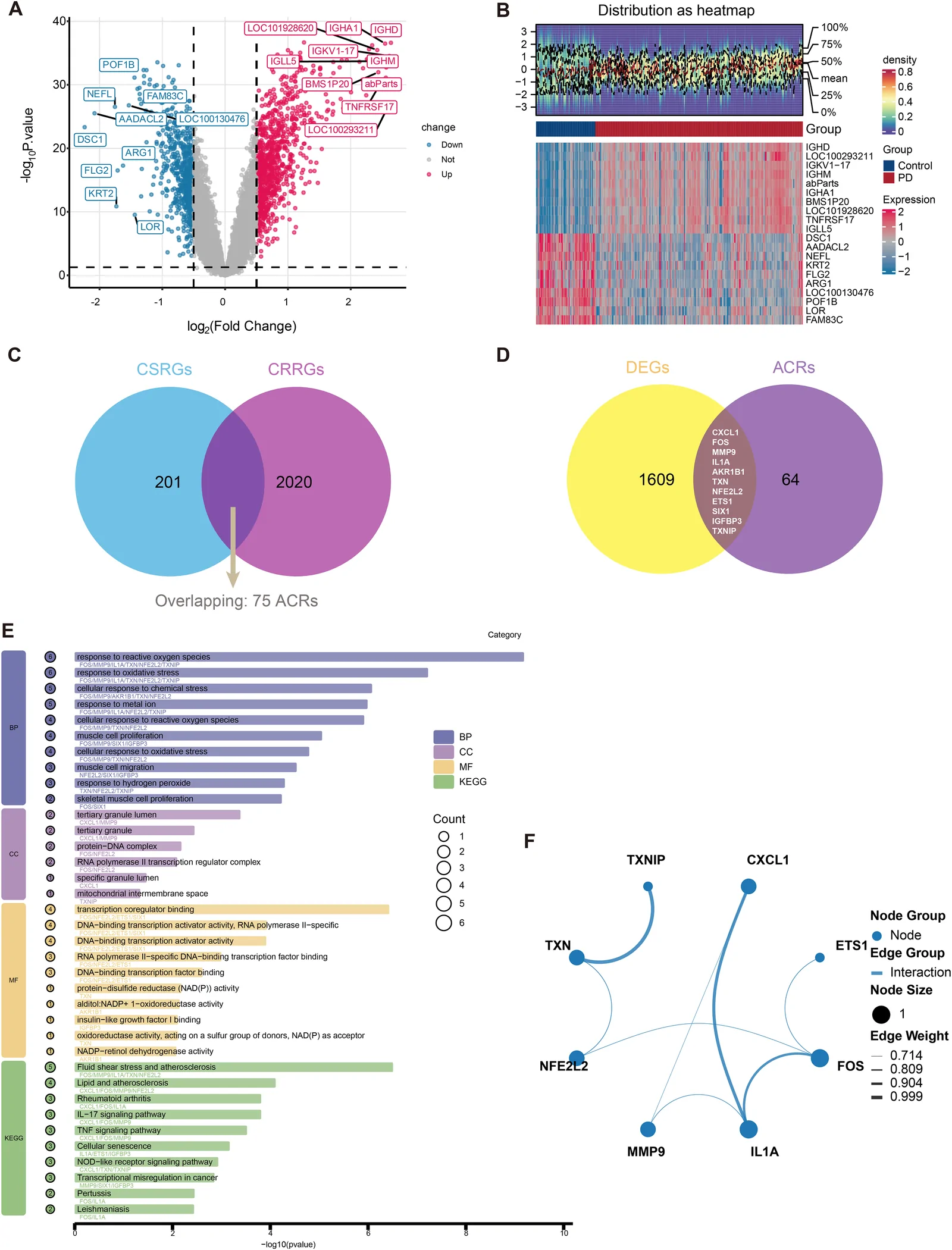
Identification of candidate genes associated with cellular senescence and circadian rhythms in periodontitis. **(A)** The volcano plot shows the differentially expressed genes in PD group compared to the control group in the GSE16134 dataset. **(B)** The heatmap shows the expression patterns of the differentially expressed genes in the PD and control groups. **(C)** The Venn diagram shows the intersection of cell senescence-related genes (CSRGs) and circadian rhythm-related genes (CRRGs). **(D)** The Venn diagram shows the intersection of differentially expressed genes (DEGs) and ACRs. **(E)** The pathways significantly enriched by candidate genes. **(F)** The protein interaction network diagram.

To evaluate the joint involvement of cellular senescence and circadian rhythms in PD, the expression of ACRs was compared with that of DEGs, yielding 11 candidate genes for subsequent analysis (**Figure 1D**, **Supplementary Table 4**). Enrichment analysis revealed 539 GO terms among these genes (*P* < 0.05; **Figure 1E**; **Supplementary Table 5**). Specifically, 494 BP terms were enriched, including responses to reactive oxygen species and oxidative stress. Six CC terms were enriched, including protein–DNA complexes and RNA polymerase II transcriptional regulatory complexes. In addition, 39 MF terms were enriched, including transcriptional coregulator binding and DNA-binding transcriptional activator activity. KEGG analysis revealed 30 significantly enriched pathways (*P* < 0.05), including fluid shear stress and atherosclerosis, lipid and atherosclerosis, and the IL-17 signaling pathway (**Figure 1E**; **Supplementary Table 6**). The PPI network contained 8 core nodes (**Figure 1F**), suggesting that these candidates may act as integrated regulatory modules in PD.

### 3.2 Characterization of *TXN* and *TXNIP* as key genes

Feature selection was performed on candidate genes using machine-learning algorithms to identify senescence- and circadian rhythm-related regulators in PD. In the LASSO model, optimal performance was obtained at the minimum lambda value of 0.0098, resulting in the identification of 8 selected genes (**Figures 2A, B**; **Supplementary Table 7**). XGBoost, Boruta, and SVM-RFE selected 11, 11, and 10 feature genes, respectively (**Figures 2C–F**; **Supplementary Tables 8–10**). Intersecting the four algorithm-derived gene sets yielded 8 feature genes: *AKR1B1*, *CXCL1*, *ETS1*, *FOS*, *IGFBP3*, *NFE2L2*, *TXN*, and *TXNIP* (**Figure 2G**). Wilcoxon rank-sum tests were subsequently used to verify genes with significant differential expression analysis was associated with both cellular senescence and circadian rhythms in PD group. Consistent with the training set, the expression differences of *TXN* and *TXNIP* were significant in the validation cohort (**Figure 2H**). Their AUC values exceeded 0.7 in both the training and validation sets (**Figures 2I, J**). Therefore, *TXN* and *TXNIP* were designated key genes and were retained for further analyses. Notably, both genes are associated with cellular senescence and circadian rhythm gene sets, suggesting that they may serve as molecular mediators that integrate these two regulatory systems in PD.

**Figure 2:**
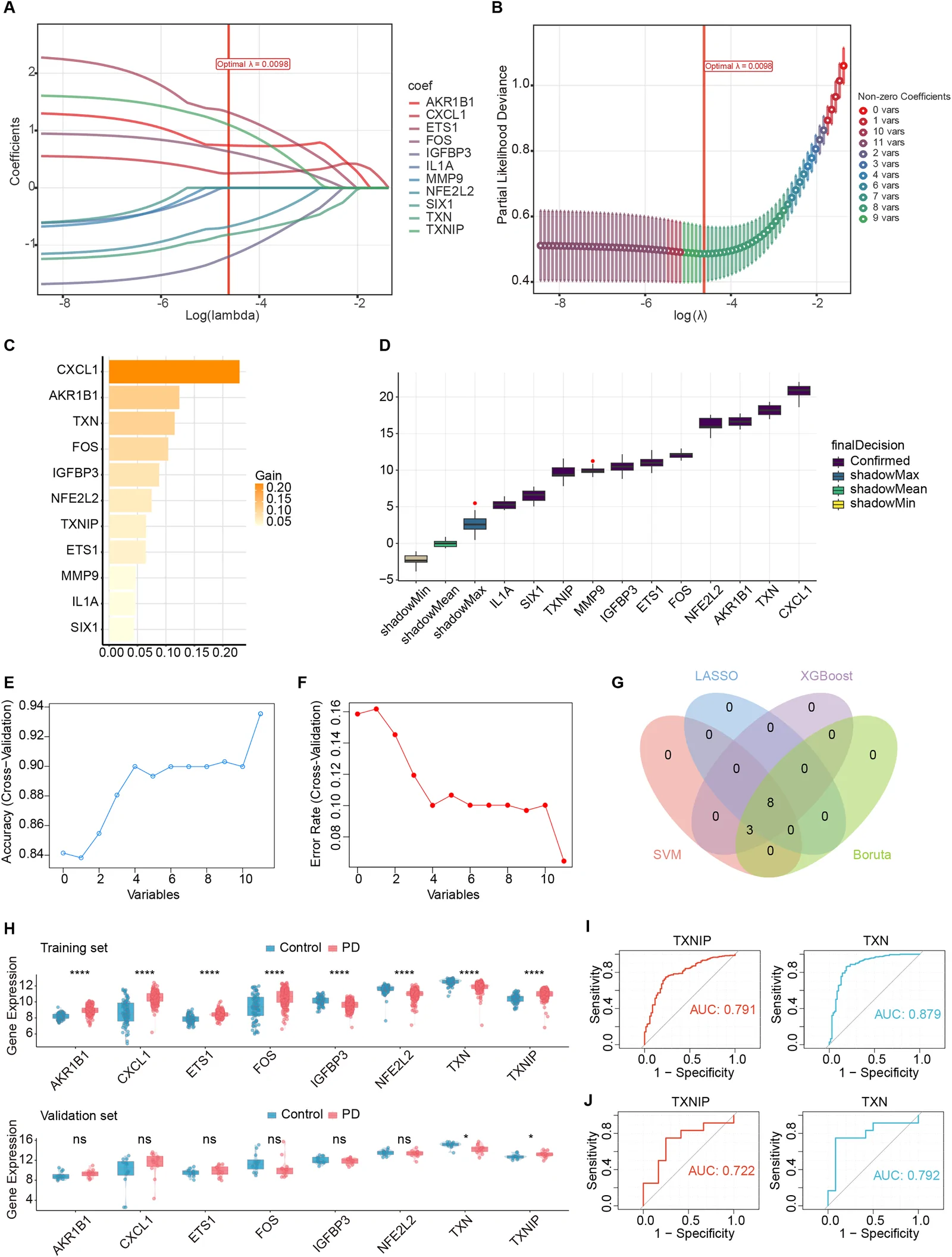
Feature gene selection based on multiple machine learning algorithms and validation of key genes. **(A)** Coefficient penalty plot of LASSO regression. **(B)** Cross-validation error curve of LASSO regression. **(C)** Feature importance plot of XGBoost algorithm. **(D)** Feature selection result plot of Boruta algorithm. **(E)** Accuracy plot of SVM-RFE algorithm. **(F)** Error rate after 10-fold cross-validation. **(G)** Venn diagram showing the intersection of the feature genes selected by LASSO, XGBoost, Boruta and SVM-RFE methods. **(H)** Boxplot of expression levels of feature genes in PD group and the control group in training set and validation set. **(I)** The ROC curve for predicting PD using *TXN* and *TXNIP* in the training set (GSE16134). **(J)** The ROC curve for predicting PD using *TXN* and *TXNIP* in the validation set (GSE173078). (* indicates *P* < 0.05, **** indicates *P* < 0.0001, ns indicates not significant).

### 3.3 Construction and validation of a nomogram of *TXN* and *TXNIP*

A nomogram based on *TXN* and *TXNIP* was generated to assess the clinical predictive value of these key genes for PD (**Figure 3A**). Calibration analysis revealed close agreement between the predicted and observed outcomes, indicating reliable model accuracy (**Figure 3B**). DCA demonstrated greater clinical net benefit for the nomogram than for the single-gene model (**Figure 3C**). The combined model also showed strong discrimination in the training set, with an AUC of 0.898 (**Figure 3D**).

**Figure 3:**
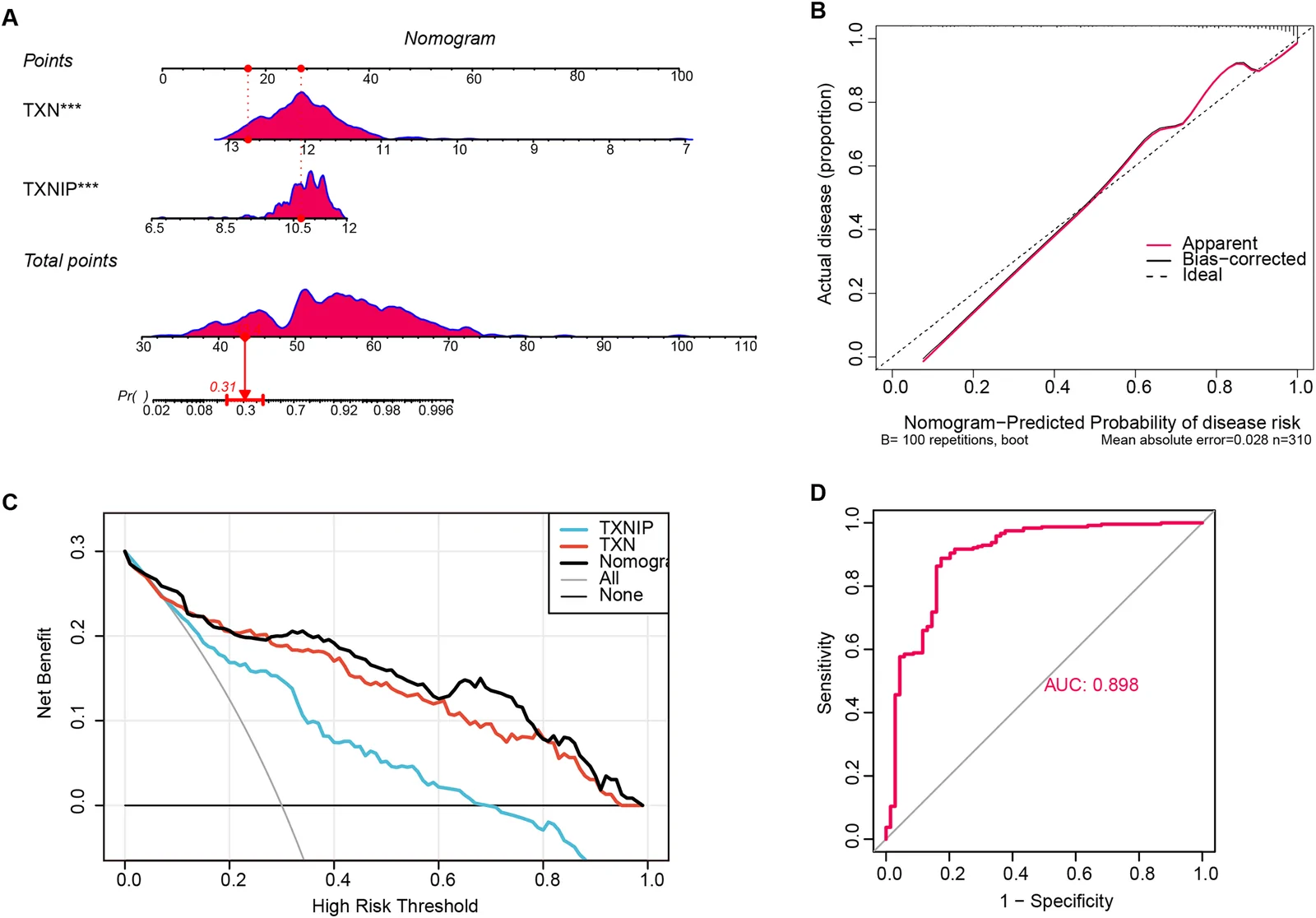
Evaluation of the predictive performance of the combined *TXN* and *TXNIP* nomogram. **(A)** A nomogram constructed based on *TXN* and *TXNIP* for predicting the risk of PD. **(B)** The calibration curve of the nomogram model. **(C)** The decision curve analysis (DCA) comparison between the nomogram model and the single-gene model. **(D)** The ROC curve of the nomogram model for predicting PD in the training set.

Chromosomal mapping placed *TXN* on chromosome 9 and *TXNIP* on chromosome 1 (**Supplementary Figure 2A**). Subcellular localization predicted that *TXN* was present in the nuclear cytoplasm and cytoplasm, whereas *TXNIP* was localized to the plasma membrane and cytoplasm (**Supplementary Figures 2B, C**). Their shared cytoplasmic localization provides spatial support for possible cooperative or interactive functions in intracellular signaling. This distribution further suggests that both *TXN* and *TXNIP* may participate in PD pathogenesis through pathways related to oxidative stress and inflammatory responses.

### 3.4 GSEA and gene set variation analysis pathway enrichment analysis of *TXN* and *TXNIP*

GSEA was performed to explore the molecular mechanisms associated with *TXN* and *TXNIP*. The analysis revealed 74 enriched pathways for *TXN* and 70 for *TXNIP* (**Figures 4A, B**; **Supplementary Tables 11, 12**). Among these pathways, 43 pathways were shared by the two genes (**Figures 4C, D**; **Supplementary Table 13**). Gene set variation analysis (GSVA) further showed that 30 of these pathways were significantly upregulated in PD (**Figure 4E**), including immune-related pathways such as B-cell receptor signaling, Toll-like receptor signaling, chemokine signaling, FcγR-mediated phagocytosis, JAK-STAT signaling, and T-cell receptor signaling (**Figure 4E**).

**Figure 4:**
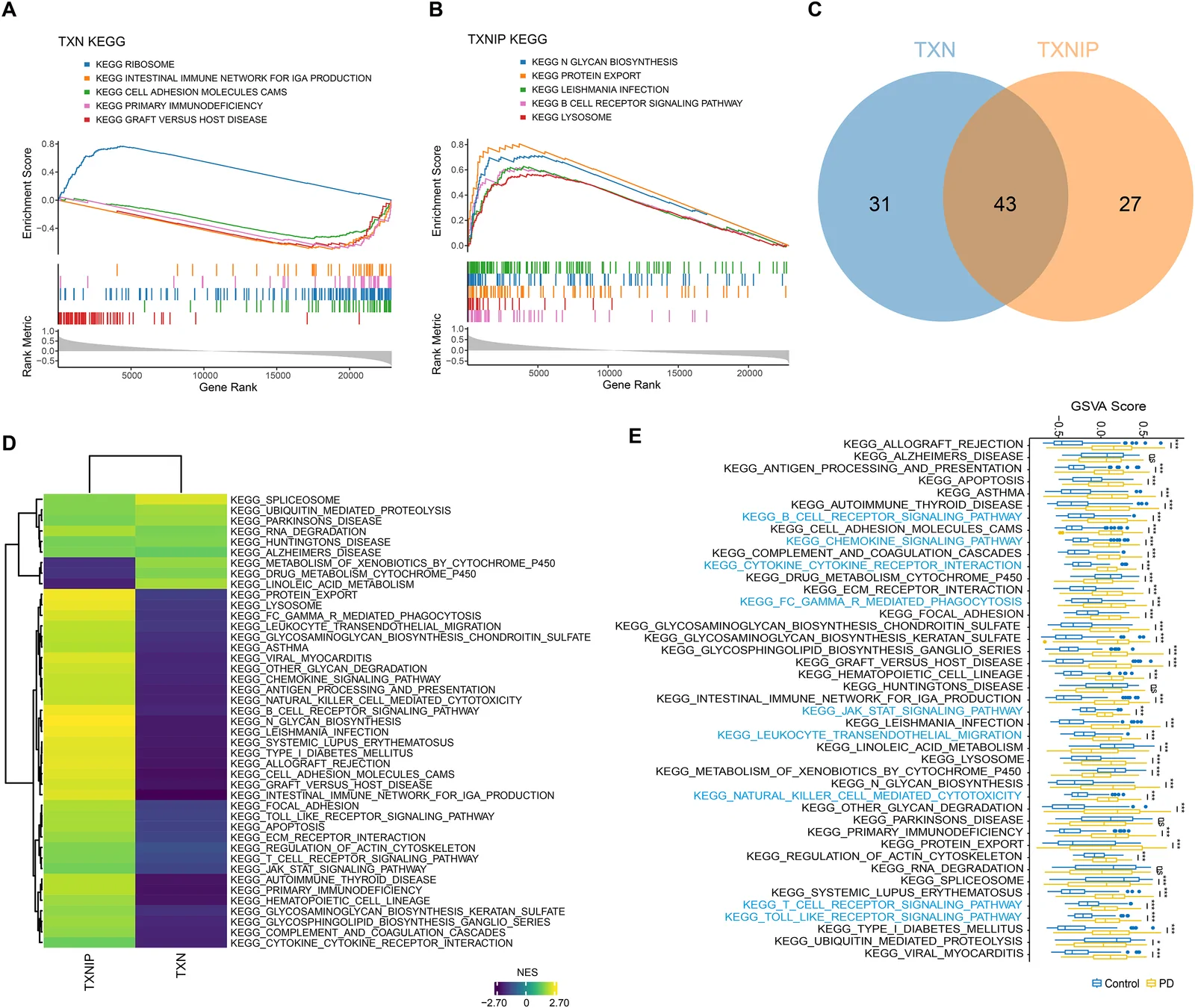
Pathway enrichment analysis of *TXN* and *TXNIP*. **(A)** The GSEA plot shows the enrichment of *TXN*-related pathways. **(B)** The GSEA plot shows the enrichment of *TXNIP*-related pathways. **(C)** The Venn diagram illustrates the overlapping enriched pathways between *TXN* and *TXNIP*. **(D)** The heatmap displays the significantly enriched pathways shared by both groups. **(E)** Differences in GSVA scores for *TXN*- and *TXNIP*-related pathways between the PD and control groups. (* indicates *P* < 0.05, *** indicates *P* < 0.001, ns indicates not significant).

### 3.5 Characteristics of immune infiltration and its correlation with *TXN*/*TXNIP* expression

Immune infiltration was next evaluated to define the PD immune microenvironment. Using the Wilcoxon rank-sum test, we identified 24 immune cell subsets whose enrichment scores significantly differed between PD and control tissues (*P* < 0.05; **Figures 5A, B**). Activated B cells, immature B cells, and activated CD4+ T cells were markedly enriched in gingival tissues from patients with PD (**Figure 5B**), indicating intensified adaptive immune activity and disturbed immune homeostasis. Spearman correlation analysis revealed that the expression of *TXN* was inversely associated with that of five immune cell types, including activated B cells, whereas the expression of *TXNIP* was positively correlated with most of these immune cell populations (**Figure 5C**). These findings indicate an active B-cell response within PD lesions.

**Figure 5:**
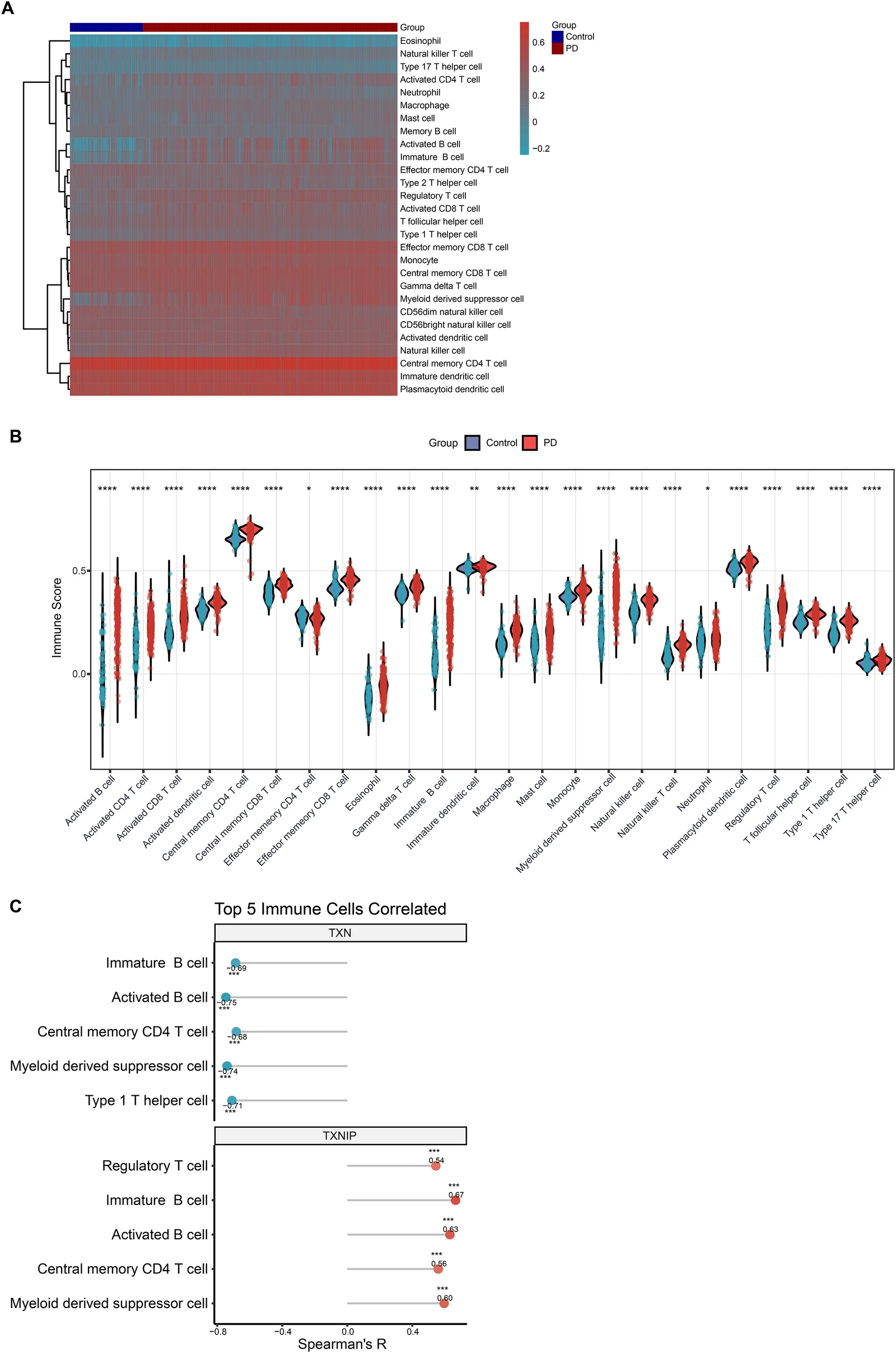
Characteristics of immune infiltration and its correlation with *TXN* and *TXNIP* expression. **(A)** The heatmap shows the single-sample gene set enrichment analysis (ssGSEA) scores for 28 immune cell types in the training set. **(B)** The box plot illustrates the 24 immune cell types with significantly different infiltration levels between the PD and control groups. **(C)** The plot displays the correlations between the key genes (*TXN*, *TXNIP*) and the differentially infiltrated immune cells. (* indicates P < 0.05, ** indicates P < 0.01, *** indicates P < 0.001, **** indicates P < 0.0001)

### 3.6 Drug prediction and construction of transcription factor and ceRNA regulatory networks

Drug prediction indicated that *TXN* directly binds gambogic acid and is also a known target of PX-12, whereas *TXNIP* showed predicted binding only to the recombinant FAS ligand (**Supplementary Figure 3A**). In the TF-mRNA network, one TF (FOXO1) was predicted for *TXN*, while eight TFs, including POLR3A and GATA2, were predicted for *TXNIP* (**Supplementary Figure 3B**). In the ceRNA network, *TXNIP* represented the main regulatory target, whereas *TXN* was linked to only one microRNA, miR-5701 (**Supplementary Figure 3C**; **Supplementary Table 14**). These results suggest that *TXN* may be a more direct and specific drug-intervention node, whereas *TXNIP* is embedded in a broader ceRNA and multi-TF regulatory architecture.

### 3.7 Single-cell and experimental analyses highlight plasma cell-associated *TXN/TXNIP* alterations in periodontitis

PCA was then performed on the single-cell dataset GSE164241. UMAP analysis combined with positive marker-gene (**Supplementary Table 15**) annotation revealed 12 cell clusters (**Figures 6A, B**). Compared with the control group, the PD group had altered immune cell proportions; for example, the proportions of both T cells and plasma cells increased (**Figure 6C**). *TXNIP* expression was significantly decreased in T cells, endothelial cells (ECs), fibroblasts (FBs), and macrophages (MACs) in PD patients, but it was elevated in plasma cells (**Figures 6D, E**). *TXN* expression was significantly downregulated in ECs, T cells, plasma cells, MACs, and other cell types in the PD group (**Figure 6E**).

**Figure 6:**
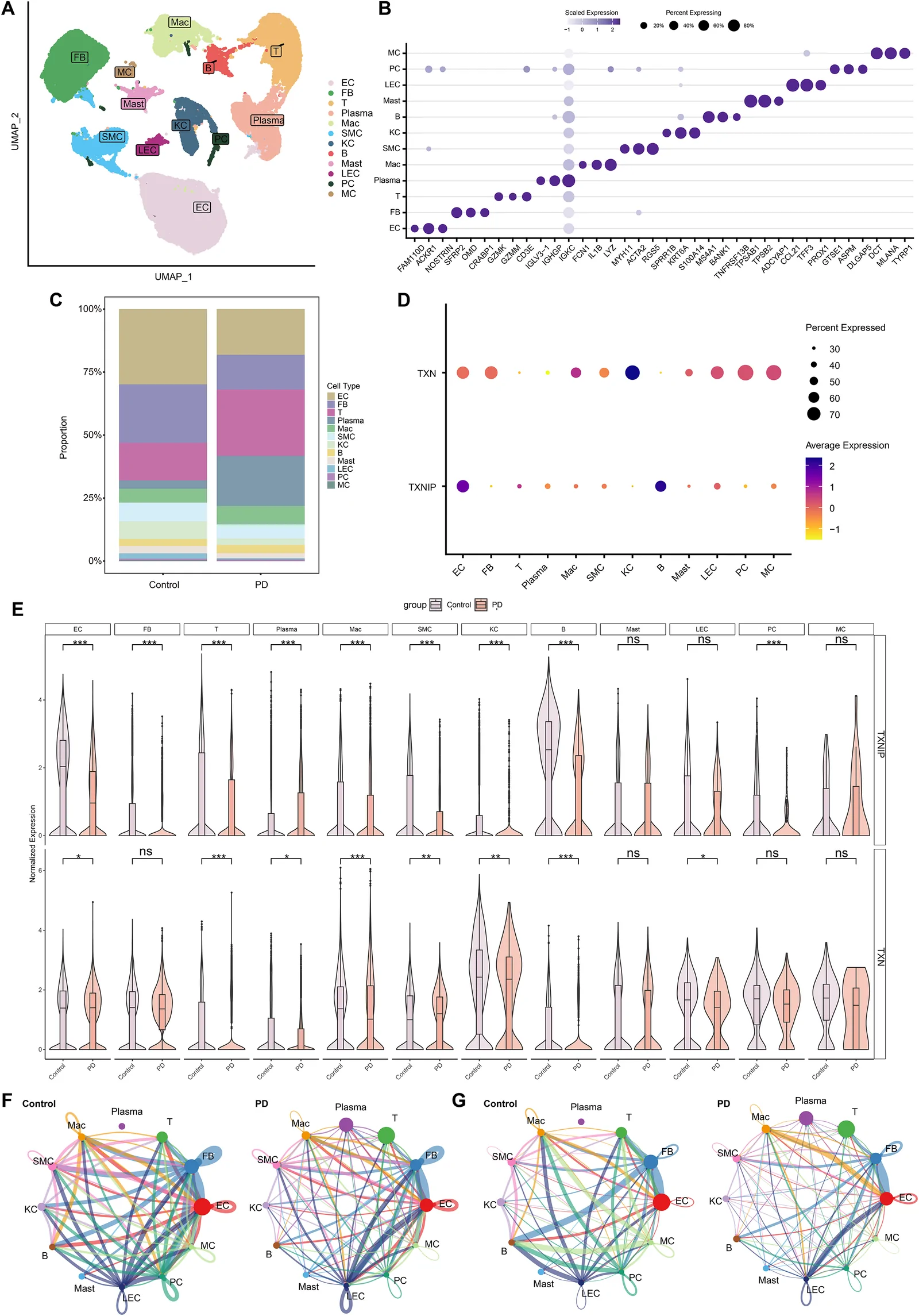
Single-cell transcriptome atlas and identification of plasma cells. **(A)** UMAP plot showing the 12 annotated cell types. **(B)** Dot plot showing marker genes of 12 annotated cell types. **(C)** Proportion distribution of annotated cell subtypes in control versus PD groups. **(D)** Expression of *TXN* and *TXNIP* in various cell types. **(E)** Expression patterns of *TXN* and *TXNIP* across different cell populations in the control and PD groups. **(F)** Network diagram of communication quantities between the control and PD groups. **(G)** Network diagram of communication weights between the control and PD groups. (* indicates *P* < 0.05, ** indicates *P* < 0.01, *** indicates *P* < 0.001, ns indicates not significant).

Cell–cell communication analysis was conducted to determine how plasma cells interact within the gingival microenvironment during PD. In the controls, communication occurred mainly among stromal populations, including FBs, ECs, and PCs, with additional macrophage-related interactions (**Figures 6F, G**). In contrast, the PD samples displayed a more complex intercellular communication network (**Supplementary Figure 4A**). No interaction between plasma cells and other cell types was detected in controls (**Figure 6F**), whereas plasma cells in PD patients actively communicated with T cells, macrophages, B cells, and lymphatic endothelial cells (LECs) (**Figure 6G**). Interactions between macrophages and endothelial cells were also enhanced under PD conditions (**Figure 6G**). Compared with that of the controls, the communication probability between plasma cells and B cells through the MIF-(CD74+CXCR4) signaling axis was increased. MIF-(CD74+CD44) signaling between plasma cells and macrophages was likewise significantly increased (**Supplementary Figure 4B**).

A mouse model of PD was established, and mice in the PD group displayed significant alveolar bone resorption (**Figure 7A**). Immunofluorescence staining revealed marked colocalization of *TXN* and *TXNIP* with CD138 (**Figures 7B, C**), indicating the expression of both genes in plasma cells. Compared with that in the controls, *TXN* expression in the PD group significantly decreased, whereas *TXNIP* expression significantly increased (**Figure 7D**). Plasma cells were therefore identified as a major cell type showing *TXN* downregulation and *TXNIP* upregulation during PD.

**Figure 7:**
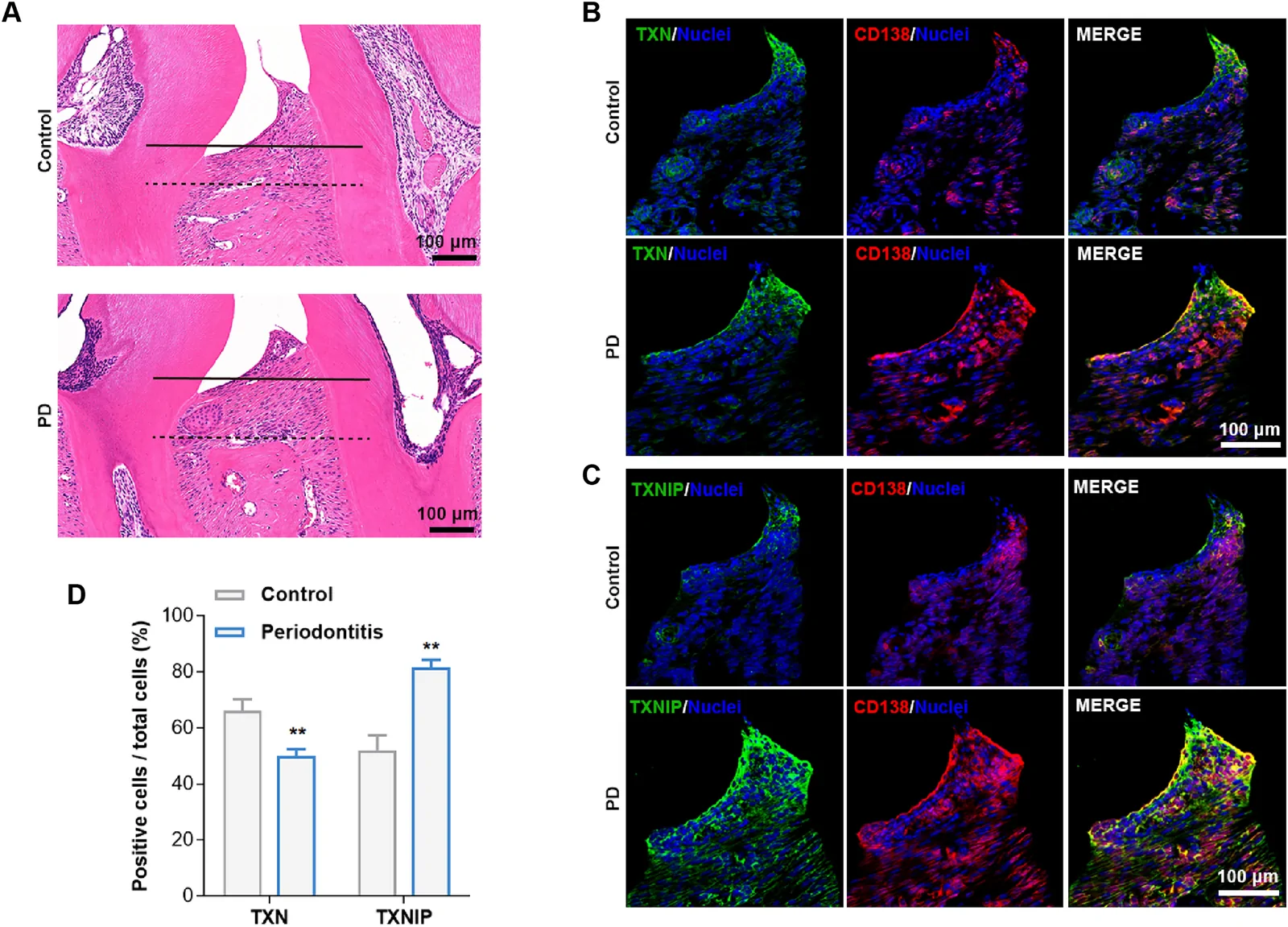
Histological and immunofluorescence validation of *TXN* and *TXNIP* expression in periodontal tissues. **(A)** H&E staining of periodontal tissues in the control and PD groups. The dashed line indicates the alveolar bone crest, and the solid line indicates the cementoenamel junction. **(B)** Immunofluorescence staining of *TXN* and CD138 in the gingiva of the control and PD groups. Green, *TXN*; red, CD138; blue, DAPI. **(C)** Immunofluorescence staining of *TXNIP* and CD138 in the gingiva of the control and PD groups. Green, *TXNIP*; red, CD138; blue, DAPI. **(D)** Statistical analysis of *TXN*- and *TXNIP*-positive cells in the control and PD groups. (*P* values were determined by two-way ANOVA test in **(D)**. ** indicates *P* < 0.01, *** indicates *P* < 0.001, ns indicates not significant).

### 3.8 Pseudotime trajectory analysis reveals the functional heterogeneity of plasma cell subtypes in periodontitis

Pseudotime trajectory analysis based on cell clustering revealed three plasma cell subtypes: Plasma_IgG4, IEG_NFKB, and Plasma_IgA_JCHAIN (**Figure 8A**). In healthy controls, plasma cells were assigned mainly to the Plasma_IgG4 subtype, whereas the IEG_NFKB and Plasma_IgA_JCHAIN subtypes increased in PD group (**Figure 8B**). Expression profiling of *TXN* and *TXNIP* along the plasma cell trajectory revealed that the expression of both genes increased overall during pseudotime progression (**Figures 8C, D**). Functional enrichment further indicated that plasma cells were enriched in selenium compound metabolism, N-linked glycosylation, chondroitin sulfate glycosaminoglycan biosynthesis, and vitamin B6 metabolism (**Figure 8E**).

**Figure 8:**
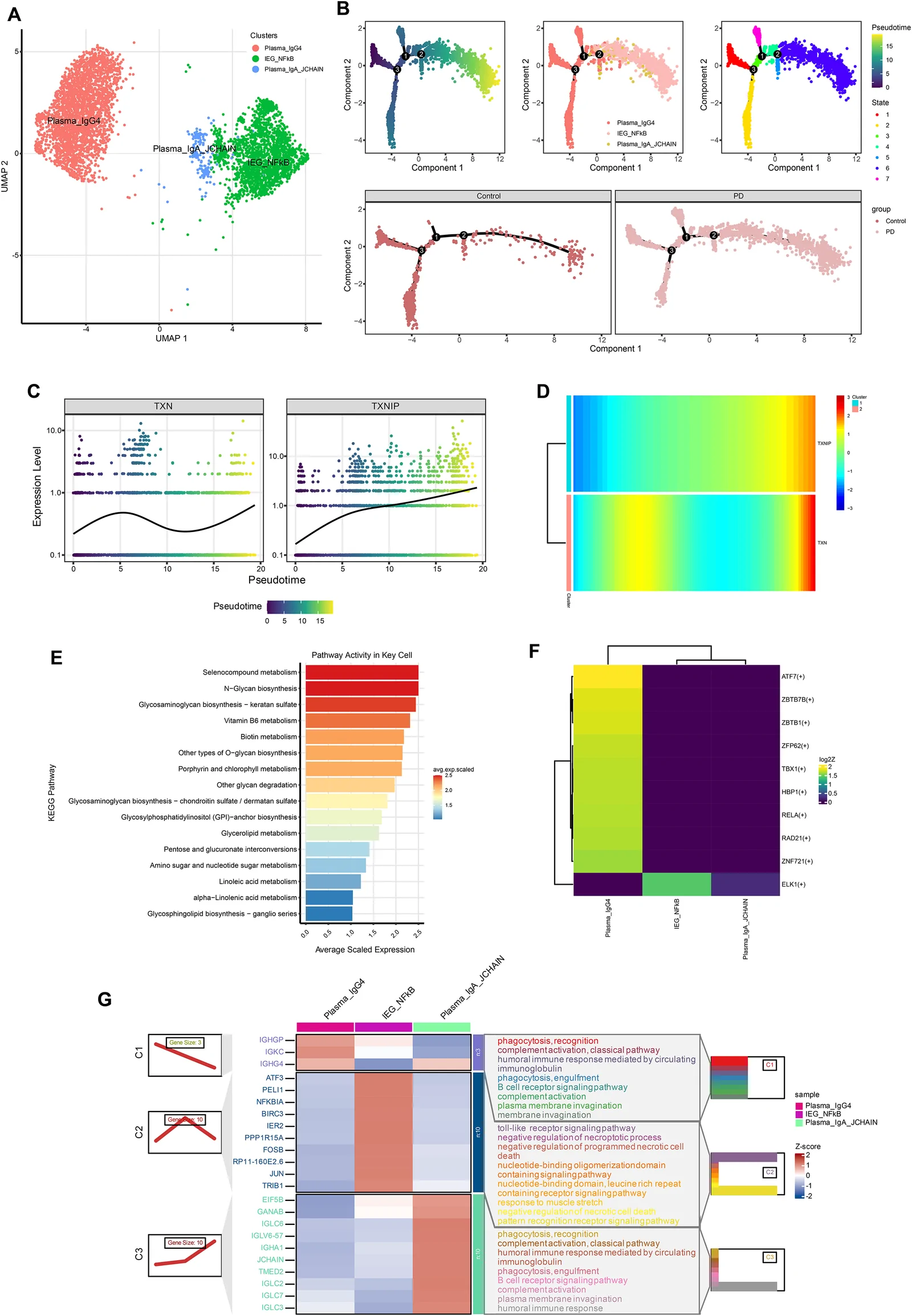
Identification of plasma cell subpopulations, differentiation trajectory, and functional characteristics. **(A)** UMAP plot showing the three distinct subpopulations of plasma cells. **(B)** Pseudotime trajectory analysis revealing the differentiation dynamics and distribution of plasma cell subpopulations. **(C, D)** Expression trends of *TXN* and *TXNIP* along the differentiation trajectory. **(E)** KEGG analysis of significantly enriched pathways in plasma cells. **(F)** Differences in specific pathway activities across the distinct plasma cell subpopulations. **(G)** Predicted transcription factors across different plasma cell subpopulations.

The plasma cell subtypes presented distinct gene expression features (**Figure 8F**). Genes highly expressed in the Plasma_IgG4 subtype, including IGHGP, IGKC, and IGHG4, were enriched in phagocytosis and complement activation and decreased along the pseudotime trajectory (**Figure 8G**). Genes highly expressed in the Plasma_IgA_JCHAIN subtype were also enriched mainly in phagocytosis and complement activation, but their expression increased during pseudotime progression (**Figure 8G**). In the IEG_NFKB subtype, highly expressed genes such as ATF3 and PELI1 were enriched mainly in the Toll-like receptor signaling pathway, whose expression tended to increase initially but then decreased along pseudotime (**Figure 8G**). Together, these results indicate functional heterogeneity among plasma cells in PD and provide a refined perspective on their role in disease progression.

## 4 Discussion

PD is a dysbiosis-driven, chronic, inflammatory, bone-destructive disease. Its progression involves multiple pathological processes, including dysregulated host immune responses, oxidative stress imbalance, and impaired tissue repair (41). However, the interactive effects and underlying molecular mechanisms of two major endogenous regulatory systems, cellular senescence and circadian rhythm, in PD remain to be systematically elucidated (42, 43). In this study, by integrating multidimensional bioinformatics analyses based on public transcriptomic and scRNA-seq datasets, we identified *TXN* and *TXNIP* as key genes associated with cellular senescence and circadian rhythm and further evaluated their potential as diagnostic biomarkers. Furthermore, through immune infiltration profiling, reconstruction of cell–cell communication networks, and single-cell pseudotime trajectory analysis, we observed that both genes are coenriched in pathways such as antigen processing and presentation. These findings may inform the development of precision diagnostic and therapeutic strategies targeting senescence and circadian rhythm regulatory pathways.

The core pathological hallmark of PD is a vicious cycle driven by oxidative stress and inflammatory responses, wherein persistent stimulation by periodontal pathogens and their metabolites triggers the overproduction of local reactive oxygen species (ROS) (44, 45). Thus, modulating excessive ROS may represent a plausible strategy for halting PD progression. Thioredoxin (*TXN*) functions as a master regulator of cellular redox homeostasis. It effectively scavenges ROS the dithiol/disulfide exchange at its catalytic center to protect cells against oxidative injury and actively participates in DNA synthesis, transcriptional regulation, and antiapoptotic signaling (46). In contrast, thioredoxin-interacting protein (*TXNIP*) is the primary endogenous repressor of *TXN*. By directly binding to the catalytic center of *TXN*, *TXNIP* abrogates its antioxidant capacity and autonomously orchestrates pathological events, including inflammasome activation, apoptosis, and metabolic derangements (47, 48). Furthermore, *TXNIP* contributes significantly to cellular senescence, with its overexpression known to promote SASP and mitochondrial dysfunction (49). From the perspective of circadian rhythms, the dysregulation of CLOCK has been shown to upregulate *TXNIP* expression, highlighting *TXNIP* as a potential convergence point between circadian control and oxidative stress (50, 51). Therefore, this pair of genes may constitute a regulatory axis potentially involved in modulating ROS accumulation and periodontal inflammation. Disruption of their expression balance is hypothesized to compromise the oxidative clearance capacity of tissue, potentially contributing to redox imbalance and periodontal ligament apoptosis, matrix degradation, and progressive alveolar bone resorption.

This study links *TXN* to the regulatory networks associated with cellular senescence and circadian rhythm, suggesting that within the pathological microenvironment of PD, *TXN* may participate in the functional remodeling of immune cells and the maintenance of periodontal tissue homeostasis through its involvement in senescence-induced oxidative stress signals with the spatiotemporal dysregulation caused by circadian disruption (52). The specific mechanisms may involve the regulation of ROS-scavenging capacity, indirect modulation of inflammasome activation, and a delay in cellular senescence through the preservation of mitochondrial function. These findings provide a novel molecular perspective for understanding the interactive “senescence–circadian rhythm–oxidative stress” network in PD. Furthermore, previous studies have demonstrated that under hypoxic conditions, *TXNIP* activates the inflammatory cascade via the ROS/*TXNIP*/NLRP3 signaling pathway, promoting the maturation and release of IL-1β and exacerbating periodontal tissue destruction (52, 53). In the PD microenvironment, microbial dysbiosis and hypoxia synergistically drive ROS accumulation, which induces *TXNIP* expression (53, 54). On the one hand, *TXNIP* compromises antioxidant defenses by binding to *TXN*; on the other hand, it activates the NLRP3 inflammasome to amplify the inflammatory response, ultimately accelerating tissue destruction by inducing cellular senescence and apoptosis. This study revealed the stable high expression of *TXNIP* in PD and correlated it with cellular senescence and circadian rhythm gene sets. These findings suggest that *TXNIP* is a potential key node that links senescence, rhythm disruption, and inflammation and is important for elucidating the pathological mechanisms and developing targeted interventions for PD. Consistent with previous reports, *TXN* serves as a key molecule in maintaining mitochondrial function; alterations in its expression can lead to the accumulation of ROS and the loss of mitochondrial membrane potential, triggering the intrinsic apoptotic pathway (46). Moreover, *TXNIP* mediates pyroptosis by activating the NLRP3 inflammasome and participates in the crosstalk between apoptosis and pyroptosis through its interaction with apoptosis-associated speck-like protein containing a CARD (ASC) (55). In summary, through interactive regulation, *TXN* and *TXNIP* translate oxidative stress signals into a three-tier cascade of inflammation amplification, cell death, and tissue destruction, serving as a critical molecular switch in the progression of PD from reversible inflammation to irreversible bone destruction.

In this study, antigen processing and presentation, along with apoptosis, were identified as crucial pathways. Notably, the apoptosis pathway ranked first among the most significantly altered pathways based on the NES, suggesting that these two pathways may mediate the effects of key genes in PD. Oxidative stress can directly impair antigen presentation capacity by damaging dendritic cell function, interfering with MHC molecule expression, and reducing the efficiency of antigenic peptide loading (56). Regarding antigen processing and presentation, our results suggest that by regulating local redox balance, *TXN* may influence the antigen presentation efficiency of antigen-presenting cells (APCs) (47). Conversely, *TXNIP*, as an activator of the NLRP3 inflammasome, may indirectly modulate the crosstalk between APCs and T cells by promoting the release of inflammatory cytokines such as IL-1β (53, 54). Furthermore, apoptosis plays a complex, dual role in PD. On the one hand, moderate apoptosis facilitates the clearance of hyperactivated inflammatory cells (e.g., neutrophils) to promote inflammation resolution (57); on the other hand, excessive apoptosis leads to the death of periodontal ligament fibroblasts, osteoblasts, and osteocytes, directly causing the destruction of periodontal supporting tissues (58). Recent studies have shown that multiple forms of programmed cell death, including apoptosis, pyroptosis, and necroptosis, extensively interact in PD, collectively constituting a composite death mode known as “PANoptosis”, which exacerbates inflammatory amplification and tissue damage (59, 60). *P.gingivalis* can trigger ZBP1-mediated macrophage PANoptosis, supporting the involvement of integrated cell-death programs in periodontal inflammatory destruction (59). In summary, by regulating the two core pathways of antigen processing and presentation and apoptosis, *TXN* and *TXNIP* translate oxidative stress signals into a cascading effect of immune dysregulation and tissue destruction.

Recent genetic studies further support the importance of immune regulation in periodontitis. Mendelian randomization identified causal associations between circulating inflammatory proteins and oral phenotypes, including S100A12 as part of an immune-mediated host–oral proteomic signature (61). Another study linked circulating immune cells to periodontitis and identified S100A9 and S100A12 as high-confidence genes involved in neutrophil-mediated disease (62). The repeated identification of S100A12 suggests a potential link between systemic immune dysregulation and periodontal tissue destruction. These findings are consistent with the immune regulatory networks involving *TXN/TXNIP* observed in our study and further support the contribution of immune-related molecules to periodontitis pathogenesis.

Profiling the immune microenvironment provides crucial information for elucidating the pathological roles of *TXN* and *TXNIP* in PD. The results of this study revealed that the expression of *TXN* was significantly negatively correlated with activated B cells, whereas that of *TXNIP* was significantly positively correlated with immature B cells, suggesting that these genes participate in remodeling the PD immune microenvironment by regulating B-cell responses. Notably, single-cell analysis revealed more refined functional remodeling of plasma cells under disease conditions. We found that the plasma cells in the control group were in a state of immunological isolation, whereas in the disease group, they extensively communicated with various cell types, including T cells, macrophages, endothelial cells, and fibroblasts. These findings indicate that plasma cells transition from a relatively isolated state to a more interactive phenotype in PD. Prior single-cell studies have indicated that B/plasma cells in PD act beyond simple antibody secretion by using ligands such as MIF to communicate with other immune cells and acquire distinct differentiation fates. Furthermore, the dynamic expression of TXN/TXNIP along the plasma cell trajectory in our study highlights plasma cells as a potential bridge between oxidative stress and local immune remodeling (13, 63). Ligand–receptor pair analysis revealed that the probability of communication between plasma cells and B-cell was highest via the MIF-(CD74+CXCR4) signaling axis, which has been confirmed to mediate B cell chemotaxis, survival, and plasma cell differentiation (64, 65). These findings emphasize that plasma cells may participate in the regulation of the local immune network through the MIF signaling axis, potentially contributing to remodeling the PD immune microenvironment. Further pseudotime trajectory analysis revealed that plasma cells undergo functional state transitions along the differentiation trajectory. Additionally, the expression of both *TXN* and *TXNIP* tended to be similar during this differentiation process, suggesting that these genes may be coregulated during plasma cell differentiation. Different plasma cell subpopulations exhibited significant differences in the activities of complement activation and Toll-like receptor signaling pathways. Furthermore, the expression of transcription factors such as ATF7 and ZBTB7B was predicted, suggesting that the functional heterogeneity of plasma cells in PD patients, and that certain subpopulations may be associated with tissue destruction, potentially through the activity of proinflammatory pathways (66, 67). In summary, the *TXN*- and *TXNIP*-associated remodeling of B-cell responses and the functional reprogramming of plasma cells collectively reveal the dynamic evolution of the PD immune microenvironment. Future studies could further target the *TXN*-*TXNIP* axis or the MIF signaling pathway to explore novel intervention strategies for restoring periodontal immune homeostasis.

Drug prediction analysis revealed potential candidate compounds for PD intervention strategies targeting *TXN* and *TXNIP*. As a direct binding target of gambogic acid and a known target of PX-12, *TXN* is regulated by only a single microRNA (miR-5701) and a single transcription factor (FOXO1), suggesting that it is a relatively linear and specific regulatory node. In the pathological process of PD, oxidative stress is a crucial driving factor. As a core member of the thioredoxin system, *TXN* is involved in maintaining the intracellular redox balance (68–70). Our drug prediction results suggest that gambogic acid (71) and PX-12 (72) may directly bind to and inhibit *TXN* activity. However, their actual effects and binding modes warrant further experimental validation. The singular nature of its regulatory network also makes *TXN* a more tractable target for precision pharmacological intervention. In contrast, *TXNIP* only shows targeted binding with recombinant FAS ligands, is regulated by eight transcription factors (such as POLR3A and GATA2), and acts as a hub in the ceRNA network. *TXNIP* is a key regulator of inflammasome activation and is closely related to host immune responses in PD. Our drug prediction results suggest that recombinant FAS ligands may act as inhibitors of the predicted interaction between *TXNIP* and proteins such as NLRP3 (73). However, this conclusion warrants further experimental validation. Therefore, the complex regulatory network of *TXNIP* suggests that a drug solely targeting *TXNIP* may be insufficient to completely block its function; combinatorial interventions targeting its upstream and downstream transcription factors or ceRNA components may be more feasible. In summary, existing drugs targeting *TXN* (gambogic acid, PX-12) may serve as candidate tool compounds for PD treatment, but targeting *TXNIP* requires combinatorial strategies involving FAS ligands or their associated network nodes.

It should be noted that the discovery analyses were based on human periodontitis samples, whereas validation was performed in mice. Human and mouse plasma cells differ in immunoglobulin subclasses, antibody affinity maturation, survival-related signaling such as the BAFF/APRIL pathway, and potentially in their abundance and infiltration patterns within periodontal tissues (74–76). Therefore, although the mouse model provided preliminary support for the observed *TXN/TXNIP* expression changes and plasma cell accumulation, the functional roles of plasma cells may not be fully equivalent between species. In addition, the 3-week mouse periodontitis model may primarily reflect an active disease state rather than chronic steady-state plasma cell behavior. Our animal experiments were intended to validate the expression trends of *TXN/TXNIP* in plasma cells rather than to directly compare plasma cell abundance or function between species. Future studies using human periodontitis tissues and multiplex immunofluorescence are needed to further validate the plasma cell-associated signaling axis and its spatial distribution in human disease.

In conclusion, we identified *TXN* and *TXNIP* as key genes related to cellular senescence and circadian rhythm, explored their expression profiles and potential functional characteristics in PD, and observed their enrichment in antigen processing and presentation and apoptosis pathways, and identified plasma cells as a relevant cell type potentially involved in their synergistic effects. Analysis of the immune microenvironment suggested that *TXN* and *TXNIP* may be linked to the disruption of immune homeostasis, potentially via their correlation with distinct B-cell subpopulations. Furthermore, under disease conditions, plasma cells transition from an immunologically isolated state to a state with more extensive intercellular interactions, interacting extensively with various cell types via the MIF signaling axis and exhibiting significant functional heterogeneity. Drug prediction screening revealed potential therapeutic compounds such as gambogic acid. However, as an exploratory bioinformatics-based study, this work primarily aimed at discovery and hypothesis generation rather than mechanistic validation, and several limitations should be acknowledged. First, the public datasets did not consistently report key clinical covariates such as periodontitis severity, age, sex, and smoking status, preventing adjustment for these factors and potentially introducing heterogeneity across datasets. Second, the drug prediction analysis was based on public databases and reflects computational associations rather than pharmacodynamic evidence. In addition, the animal experiments were limited to validating the expression and cellular localization of *TXN* and *TXNIP* without interventions for gaining or losing function. The mechanisms underlying the opposite expression patterns of *TXN* and *TXNIP* in plasma cells also remain unclear. Future studies should incorporate independent prospective cohorts with complete clinical information, functional experiments to clarify the biological roles of *TXN* and *TXNIP*, pharmacological validation of candidate compounds, and multiplex immunofluorescence in human periodontitis tissues to confirm the spatial distribution of the plasma cell-associated signaling axis.

## Data Availability

The datasets presented in this study can be found in online repositories. The names of the repository/repositories and accession number(s) can be found in the article/**Supplementary Material**.
