# Supplementary material for: TXN/TXNIP signatures point to plasma cells as a potential immune hub in periodontitis based on integrated transcriptomic analysis and preliminary experimental validation
Source: Front Immunol. 2026 Sep 30;17:1937134. doi: 10.3389/fimmu.2026.1937134 (PMC13661806; doi:10.3389/fimmu.2026.1937134)
Supplement: Supplementary file 1 [file Image1.pdf]

TXN/TXNIP Signatures Reveal Plasma Cells as an Immune Hub in Periodontitis

This file contains Supplementary Figures 1-4.

Supplementary figure legends

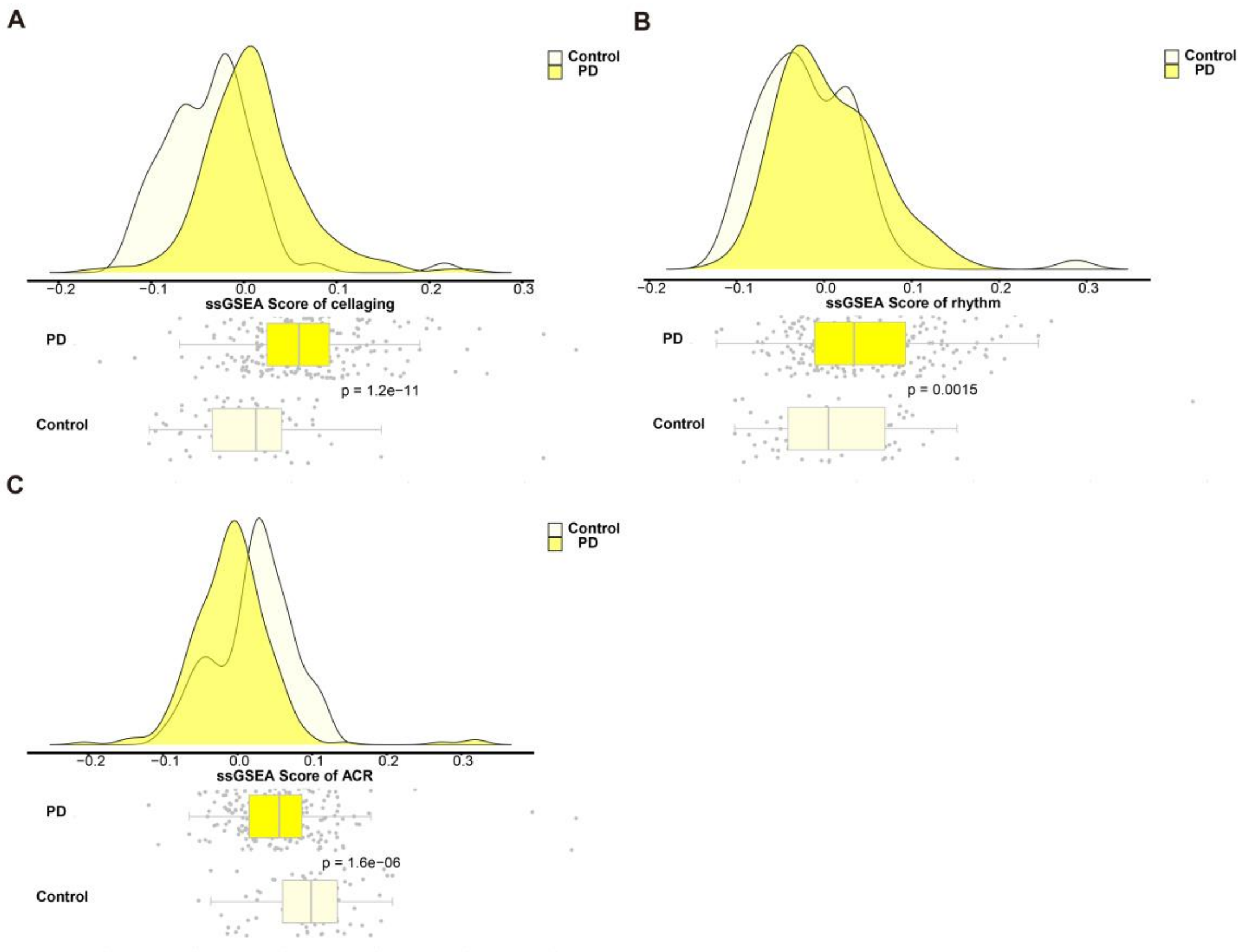

Supplementary Figure 1. Enrichment of senescence- and circadian rhythm-related gene sets in PD tissues.

ssGSEA scores of CSRGs (A), CRRGs (B), and ACRs (C) in PD versus control samples from GSE16134. Box plots display the median, IQR, and range. Significance was assessed by Wilcoxon rank-sum test.

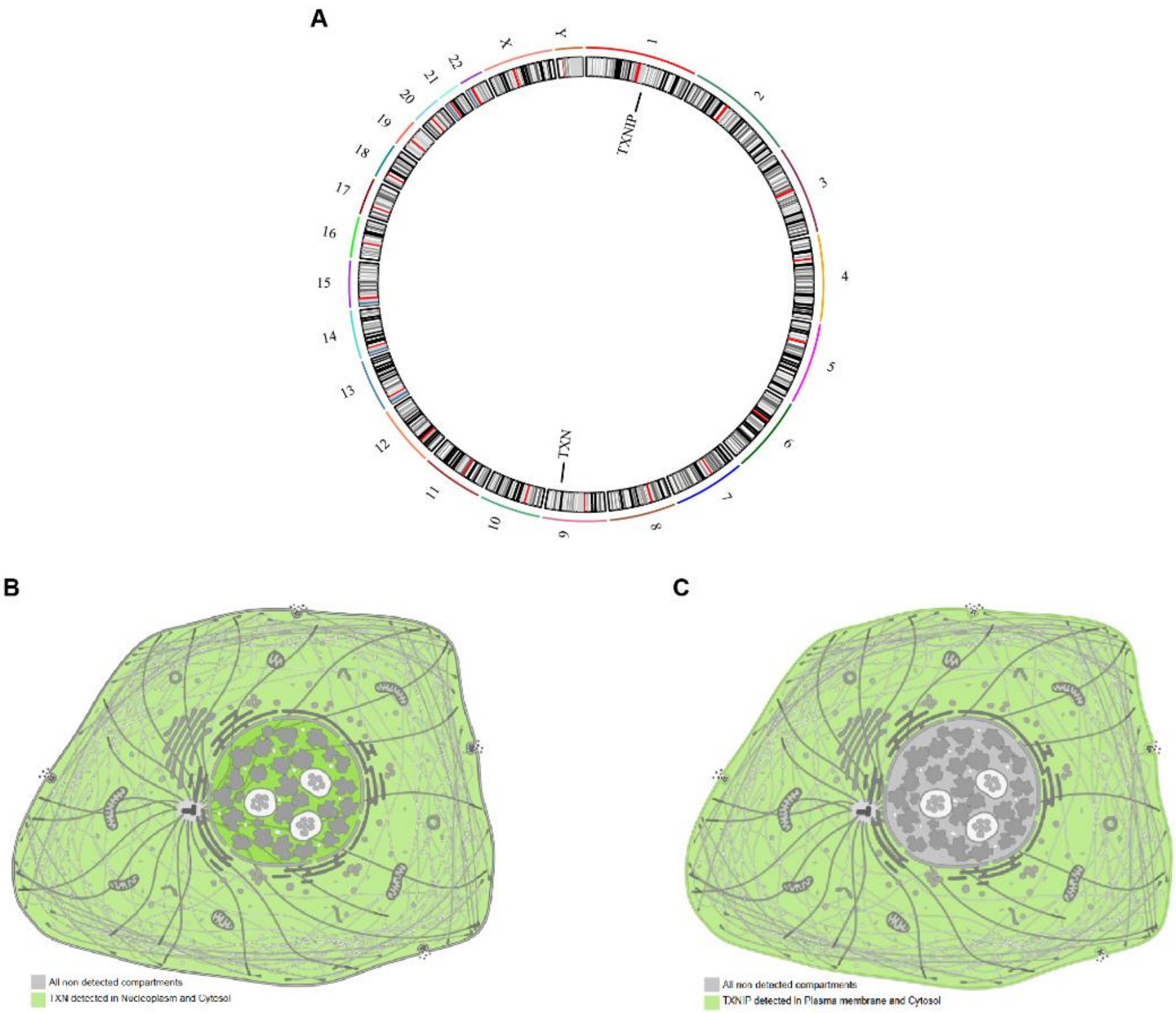

Supplementary Figure 2. Chromosomal and subcellular localization analysis of *TXN* and *TXNIP*.

(A) The chromosome mapping shows the distribution positions of the *TXN* and *TXNIP* genes on the human chromosomes.

(B) The subcellular localization map shows the distribution of *TXN* within the cell.

(C) The subcellular localization map shows the distribution of *TXNIP* within the cell.

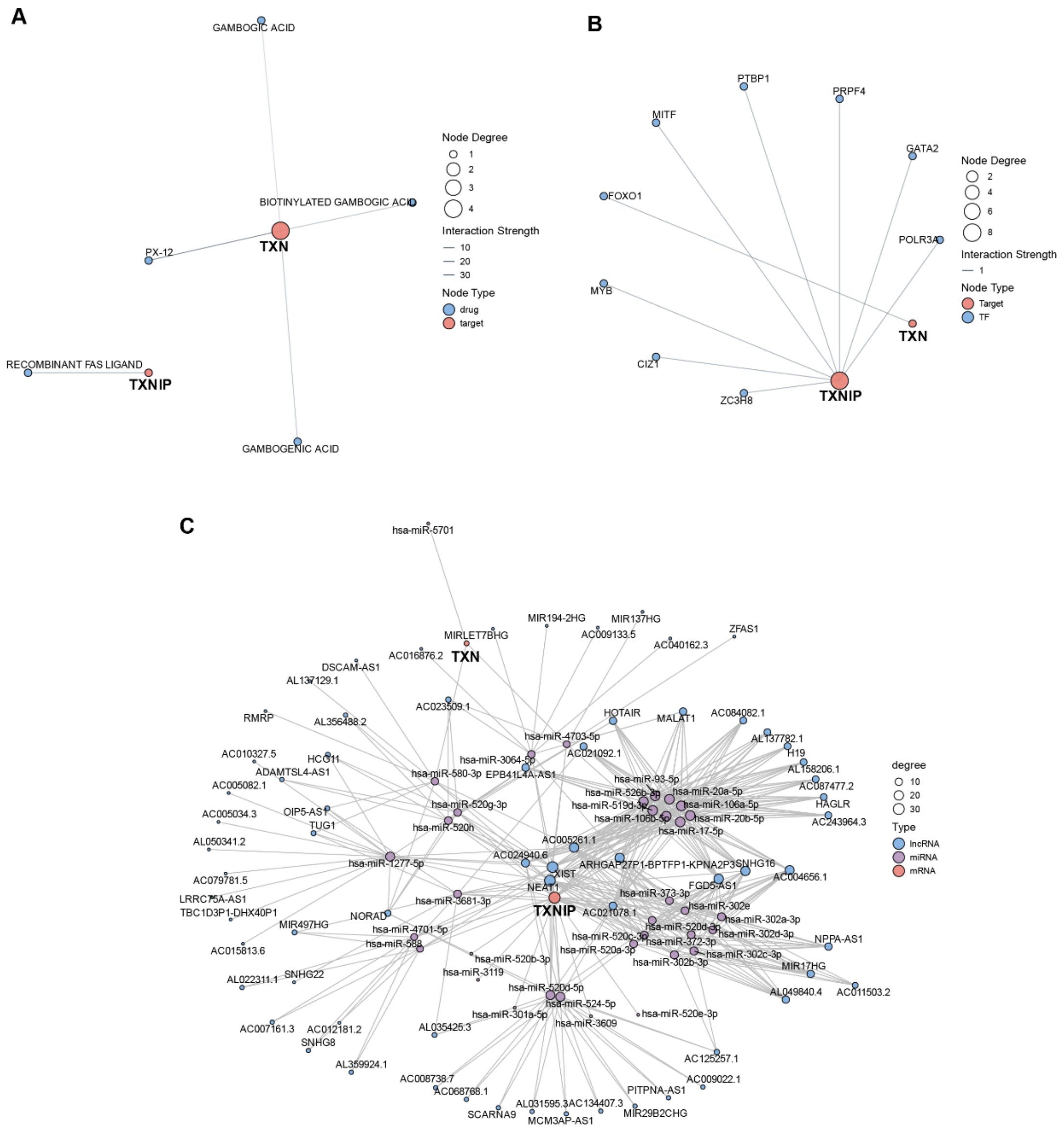

Supplementary Figure 3. Drug prediction and construction of transcription factor and ceRNA regulatory networks.

(A) Candidate drugs predicted based on the DsigDB database that interact with *TXN* and *TXNIP*.

(B) Transcription factor-gene regulatory network diagram showing the transcription factors (TFs) interacting with *TXN* and *TXNIP*.

(C) ceRNA regulatory network diagram showing the interaction relationships between miRNAs related to *TXN* and *TXNIP* and their upstream lncRNAs.

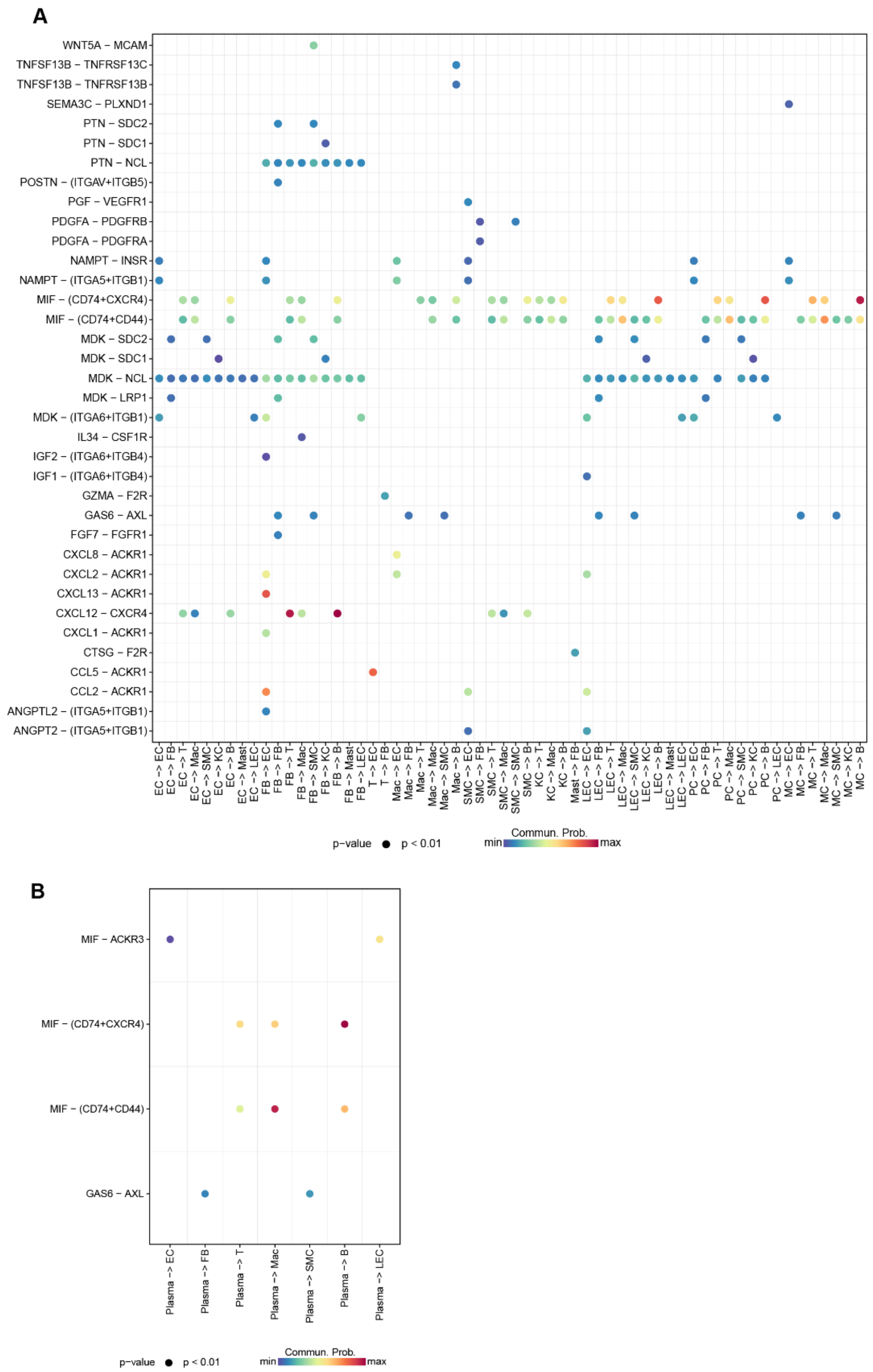

Supplementary Figure 4. Cell-cell interactions and plasma cell-specific communication networks in periodontitis.

(A) Receptor-ligand communication networks among cell types in the PD and control groups.

(B) Receptor-ligand communication networks involving plasma cells in the PD and control groups.
